# Temporal Study of *Salmonella enterica* Serovars Isolated from Environmental Samples from Ontario Poultry Breeder Flocks between 2009 and 2018

**DOI:** 10.3390/pathogens12020278

**Published:** 2023-02-08

**Authors:** Carolyn E. Murray, Csaba Varga, Rachel Ouckama, Michele T. Guerin

**Affiliations:** 1 Department of Population Medicine, Ontario Veterinary College, University of Guelph, Guelph, ON N1G 2W1, Canada; 2 Department of Pathobiology, College of Veterinary Medicine, University of Illinois at Urbana-Champaign, Urbana, IL 61802, USA; 3 Maple Lodge Hatcheries Ltd., Port Hope, ON L1A 3V5, Canada

**Keywords:** *Salmonella enterica*, monitoring, poultry, breeder flocks, temporal cluster, public health, Ontario, Canada

## Abstract

This study’s goal was to determine the prevalence, temporal trends, seasonal patterns, and temporal clustering of *Salmonella enterica* isolated from environmental samples from Ontario’s poultry breeding flocks between 2009 and 2018. Clusters of common serovars and those of human health concern were identified using a scan statistic. The period prevalence of *S. enterica* was 25.3% in broiler breeders, 6.4% in layer breeders, and 28.6% in turkey breeders. An overall decreasing trend in *S. enterica* prevalence was identified in broiler breeders (from 27.8% in 2009 to 22.1% in 2018) and layer breeders (from 15.4% to 4.9%), while an increasing trend was identified in turkey breeders (from 12.0% to 24.5%). The most common serovars varied by commodity. Among broiler breeders, *S. enterica* serovars Kentucky (42.4% of 682 submissions), Heidelberg (19.2%), and Typhimurium (5.4%) were the most common. *Salmonella enterica* serovars Thompson (20.0% of 195 submissions) and Infantis (16.4%) were most common among layer breeders, and *S. enterica* serovars Schwarzengrund (23.6% of 1368 submissions), Senftenberg (12.9%), and Heidelberg and Uganda (9.6% each) were most common among turkey breeders. *Salmonella enterica* ser. Enteritidis prevalence was highest in submissions from broiler breeders (3.7% of 682 broiler breeder submissions). Temporal clusters of *S. enterica* serovars were identified for all poultry commodities. Seasonal effects varied by commodity, with most peaks occurring in the fall. Our study provides information on the prevalence and temporality of *S. enterica* serovars within Ontario’s poultry breeder flocks that might guide prevention and control programs at the breeder level.

## 1. Introduction

Foodborne salmonellosis is a significant public health issue in many countries, including Canada. Historically, 6000 to 8000 cases of human illness caused by non-typhoidal *Salmonella enterica* are reported each year in Canada [1]. The province of Ontario had 18.7 cases per 100,000 persons in 2018 [2]; this figure is slightly lower than the national incidence rate of 19.3 cases per 100,000 persons in the same year [2]. With considerations for lost work, medical care, and economic losses to food companies and restaurants, the estimated economic burden of salmonellosis in Canada is CAD 1 billion annually [3].

Many *S. enterica* serovars can cause disease in humans. Those of most significant concern to human health are *S. enterica* serovars Enteritidis, Heidelberg, and Typhimurium, accounting for more than 50% of all reported human salmonellosis cases in Ontario and Canada in 2017 [4,5]. Consumption of contaminated poultry products (such as meat or eggs), milk, cheese, and fresh produce, and direct contact with pet turtles, hedgehogs, and chicks are associated with most cases of salmonellosis in humans [6,7,8,9]. In 2011, 63% of Canadians’ non-typhoidal salmonellosis cases were attributed to consuming contaminated poultry products [6,10,11].

Both horizontal and vertical transmission is important in *S. enterica* contamination of poultry hatching eggs, and, consequently, poultry hatcheries. The horizontal transmission includes the indirect transfer of *S. enterica* through the environment, via transportation equipment, or by vectors, such as rodents or red mites [12,13]. In vertical transmission, specific serovars (primarily *S. enterica* serovars Enteritidis, Typhimurium, and Heidelberg) transfer directly in ovo from a colonized breeder hen to her progeny [6,10,11]. The hatchery environment is a crucial component in the control of *S. enterica* within commercial flocks, as newly hatched birds can be exposed to *S. enterica* in the hatchery and carry it with them to commercial farms.

The Ontario Hatchery and Supply Flock Policy is a monitoring program established to detect *S. enterica* serovars Gallinarum and Pullorum (host-adapted serovars), as well as *Mycoplasma* spp., in poultry breeder flocks in Ontario [14]. Scheduled testing is conducted on environmental samples collected from all domestic breeder flocks, including broiler breeders, layer breeders, and turkey breeders. Environmental testing of flocks is optional for other poultry commodities (waterfowl breeders and game bird breeders). Although the program was initiated to detect the host-adapted serovars, all *S. enterica* serovars are captured through this program.

Our research builds on previous research conducted in Ontario to assess the temporal trends of *S. enterica* serovars in poultry breeder flocks between 1998 and 2008 [15]. Using data collected as part of the Ontario Hatchery and Supply Flock Policy, the objectives of this study were to (i) determine the period prevalence of *S. enterica* (all serovars) in Ontario’s breeder flocks between 2009 and 2018, (ii) identify the most commonly isolated *S. enterica* serovars for each poultry commodity, (iii) examine the overall and serovar-specific long-term trends and seasonal patterns of *S. enterica* for each poultry commodity, and (iv) identify temporal clusters of *S. enterica* serovars.

## 2. Materials and Methods

Monitoring data from domestic breeder flocks registered under the Ontario Hatchery and Supply Flock Policy between 2009 and 2018 were obtained from the Animal Health Laboratory, University of Guelph, Guelph, Ontario. Under this program, samples are collected a minimum of three times throughout the flock’s life. For the Multiplier Monitoring Program, the flock is sampled at 1 day of age (chick box papers), 16–24 weeks of age or 2–4 weeks before transfer (swab samples), and once at the peak of production (swab samples) (Table 1). For certification, additional samples are collected during rearing and/or production, depending on the program. One swab sample is collected per 1000 birds in the flock or a minimum of three samples per flock. Environmental samples include swabs from water lines and nests, litter, and fresh feces.

All samples submitted to the Animal Health Laboratory were cultured for *S. enterica* following the lab’s standard operating procedures. The Animal Health Laboratory is a diagnostic facility accredited by the American Association of Veterinary Laboratory Diagnosticians, which operates as Ontario’s provincial animal health lab. Environmental swabs were immersed in 100 mL of buffered peptone water and incubated for 24 h at 35 °C. One hundred microliters of the suspension were then inoculated on three equally spaced spots on modified, semi-solid Rappaport–Vassiliadis (MSRV) agar and incubated for 24 and 48 h at 42 °C to detect motile salmonellae. After 24 and 48 h (if negative at 24 h) incubation, MSRV plates with an opaque turbid area around the inoculation spots were used to stab a 1 μL loop at the edge of the turbid area, and this material was transferred to selective brilliant green sulfa-novobiocin (BGS-N) and xylose lysine tergitol-4 (XLT-4) agar plates. These plates were then incubated at 35 °C and examined at 24 and 48 h. Presumptive *Salmonella* spp. colonies (i.e., pink to red, with or without a black center) were confirmed by using matrix-assisted laser desorption ionization time-of-flight mass spectrometry (MALDI-TOF) (Bruker Ltd., Billerica, MA, USA) using one suspicious colony per plate if colonies were morphologically identical, or multiple colonies if different morphologies were present. Serotyping of isolates was conducted at the OIE (World Organisation for Animal Health) *Salmonella* reference laboratory at the National Microbiology Laboratory in Guelph, using the Kauffmann–White–Le Minor classification scheme. The reference laboratory discontinued phage typing of isolates after August 2017.

The dataset from the Animal Health Laboratory contained the submission identification, sample identification, submission date, client identification and city, poultry commodity (see below), sample type (chick box paper, swab sample, or boot sample), culture results, and serovar(s) and phage type(s) isolated. No unique identifier was included within the dataset for flock identification. Culture results that yielded no *S. enterica* growth or were positive for other bacteria (e.g., *Pseudomonas* spp.) were considered negative for *S. enterica* and retained in the dataset. Each submission consisted of three or more environmental samples collected from the same flock on the same day. Duplicate serovars and phage types from the same submission were removed from the dataset. However, if different serovars were isolated from the same submission, each serovar was retained in the dataset. A submission was considered *S. enterica*-positive if at least one sample tested positive for *S. enterica*, regardless of the number of serovars isolated from the sample, and *S. enterica*-negative if none of the samples tested positive for *S. enterica*.

### 2.1. Descriptive Analysis

Descriptive analyses were conducted using Microsoft Office Excel 2016 (Microsoft Corporation, Redmond, WA, USA) and Stata IC version 16 (STATA Corporation, College Station, TX, USA). Commodities were grouped as broiler breeders, layer breeders, turkey breeders, waterfowl breeders (ducks and geese), and game bird breeders (partridges, pheasants, and quail). The number of environmental samples submitted, the number of submissions, and the number of *S. enterica*-positive submissions (defined above) were tabulated for each commodity. The overall, submission-level, period prevalence of *S. enterica* for the major commodities (broiler breeders, layer breeders, and turkey breeders) was estimated by dividing the number of *S. enterica*-positive submissions by the total number of submissions for the commodity between 2009 and 2018 and multiplying by 100. Exact binomial 95% confidence intervals for the prevalence estimates were calculated.

The annual submission-level prevalence of *S. enterica* for the major commodities was estimated by dividing the number of *S. enterica*-positive submissions for each year by the total number of submissions from the same year from the same commodity and multiplying by 100. The long-term trends in the submission-level prevalence were depicted graphically.

The seasonal submission-level prevalence of *S. enterica* (overall and for all serovars with at least 100 submissions during the study period) for the major commodities was estimated by dividing the number of *S. enterica*-positive submissions for each season by the total number of submissions from the same season from the same commodity and multiplying by 100. In keeping with previous Canadian studies [15,16,17,18], winter was defined as January to March, spring as April to June, summer as July to September, and fall as October to December. The seasonal patterns in the submission-level prevalence were depicted graphically.

To identify the most common *S. enterica* serovars (overall and commodity-specific), the frequency with which each serovar was isolated was tabulated for the major commodities. For this purpose, a submission could be counted more than once because some submissions tested positive for more than one serovar. The long-term trends in the prevalence of the most common *S. enterica* serovars were illustrated graphically.

### 2.2. Temporal Cluster Detection

A retrospective, temporal scan statistic was utilized to identify periods of higher-than-expected prevalence of *S. enterica* serovars using SaTScan software version 9.6 [19]. For each major commodity, cluster detection was conducted for all serovars with a frequency of at least 20 isolates during the study period. For this purpose, a case was defined as a submission positive for a specific serovar within a particular commodity, during a particular period. A non-case was defined as a submission positive for a different serovar or an *S. enterica*-negative submission within the same commodity and period. For instance, an *S. enterica*-negative submission and a submission positive for *S. enterica* ser. Heidelberg would both be considered negative for *S. enterica* ser. Typhimurium. The smallest time unit was represented by the month and year of *S. enterica* testing.

A Bernoulli model was used to estimate the relative risk and log-likelihood ratio. This model was selected because the data consisted of two possible outcomes—cases and non-cases. The model compares the proportion of cases of a specific serovar within the time window to the proportion of cases of that same serovar outside the time window. In keeping with previous studies [15,16,17,18], the temporal scan statistic’s default 50% scanning window was chosen to examine every possible period within the study period.

The significance of each temporal cluster was assessed using a likelihood ratio test statistic; the statistic reflects the difference between the number of observed cases and the number expected under the null hypothesis of no temporal trend. A simulated *p*-value of ≤0.05, calculated through a Monte Carlo simulation using 999 replications, signified that the cluster was significant. The iterative scan option was used to identify a primary cluster (highest likelihood ratio) and all possible significant secondary clusters.

## 3. Results

Overall, 108,681 environmental samples from all poultry commodities were submitted to the Animal Health Laboratory between 2009 and 2018. There were only a few samples from waterfowl breeders and game bird breeders (146 and 161, respectively). These samples were excluded from the analyses because they were not considered representative of the waterfowl breeder and game bird breeder populations; thus, 108,374 samples from 8009 submissions from the major commodities were included in the analyses. Of 8009 submissions, 1802 (22.5%) were *S. enterica*-positive (Table 2).

The frequency distribution of *S. enterica* serovars among submissions during the 10-year study period is presented in Table 3. Overall, 97 different serovars were isolated, although many occurred infrequently. In total, 443 of 1802 *S. enterica*-positive submissions had more than one serovar, totaling 2245 submissions with unique serovars. The five most commonly isolated serovars were *S. enterica* ser. Schwarzengrund (14.9% of 2245 positive submissions), *S. enterica* ser. Kentucky (13.2%), *S. enterica* ser. Heidelberg (11.8%), *S. enterica* ser. Senftenberg (8.5%), and *S. enterica* ser. Uganda (5.9%). Three of these were isolated almost exclusively from turkey breeders: *S. enterica* serovars Schwarzengrund (96.7% of 334 positive submissions), Senftenberg (92.1% of 191 submissions), and Uganda (100% of 132 positive submissions). *Salmonella enterica* ser. Kentucky was isolated almost exclusively from broiler breeders (97.3% of 297 positive submissions). *Salmonella enterica* ser. Heidelberg was isolated mainly from turkey breeders and broiler breeders (49.6% and 49.2% of 266 positive submissions, respectively). *Salmonella enterica* ser. Enteritidis was isolated mainly from broiler breeders (86.2% of 29 positive submissions), although overall, it was isolated infrequently (1.3% of 2245 positive submissions).

### 3.1. Broiler Breeders

#### 3.1.1. Prevalence, Trends, and Seasonal Patterns

**Figure 1:**
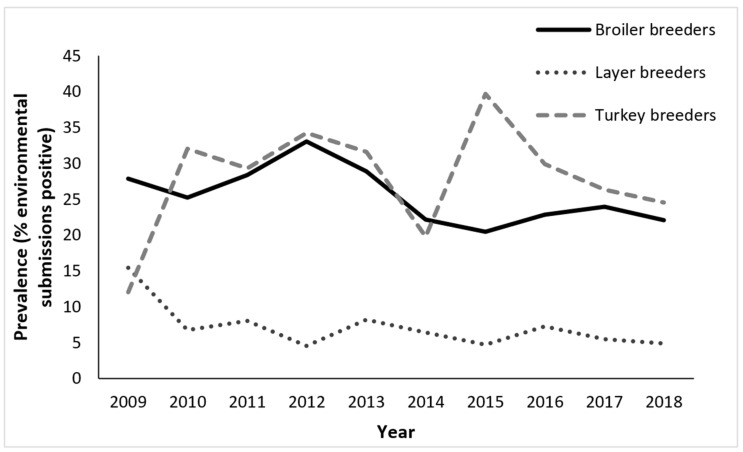
Trends in the submission-level prevalence of *Salmonella enterica* isolated from environmental samples submitted through the Ontario Hatchery and Supply Flock Policy between 2009 and 2018, by major poultry commodities. Broiler breeders (*n* = 2446 submissions); Layer breeders (*n* = 1848 submissions); and Turkey breeders (*n* = 3715 submissions). A submission was considered *S. enterica*-positive if at least one sample tested positive for *S. enterica*, regardless of the number of serovars isolated from the sample, and *S. enterica*-negative if none of the samples tested positive for *S. enterica*.

**Figure 2:**
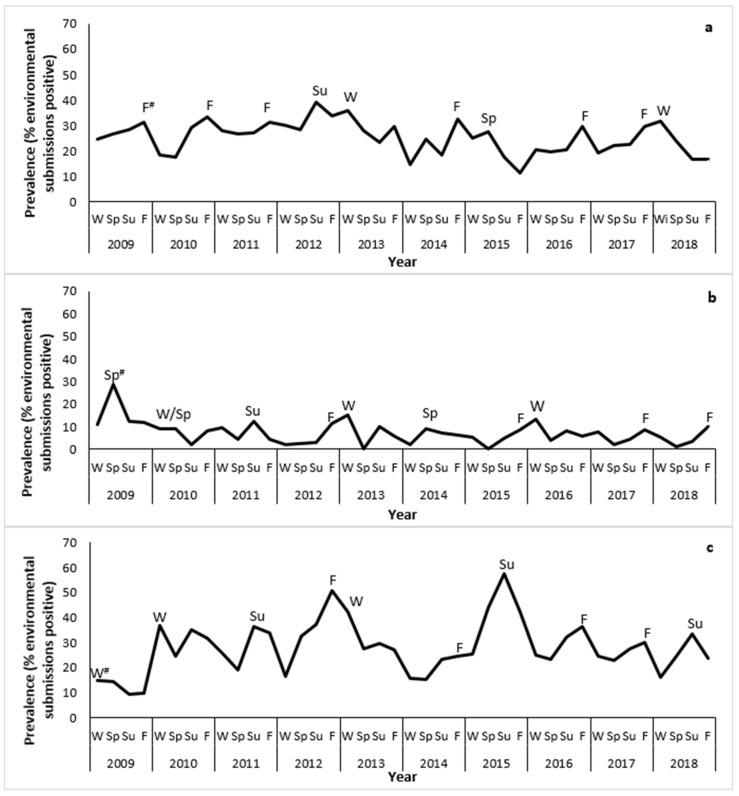
Seasonal patterns in the submission-level prevalence of *Salmonella enterica* isolated from environmental samples submitted through the Ontario Hatchery and Supply Flock Policy between 2009 and 2018, by major poultry commodities. (**a**) Broiler breeders; (**b**) Layer breeders; and (**c**) Turkey breeders. W = Winter; Sp = Spring; Su = Summer; and F = Fall. A submission was considered *S. enterica*-positive if at least one sample tested positive for *S. enterica*, regardless of the number of serovars isolated from the sample, and *S. enterica*-negative if none of the samples tested positive for *S. enterica*. # Denotes the season with the highest prevalence each year.

There were 2446 submissions from broiler breeders between 2009 and 2018 (Table 2). Of those, 619 (25.3%, 95% CI: 23.6 to 27.0%) tested positive for *S. enterica*. Overall, there was a slightly decreasing trend in the submission-level prevalence during the study period (from 27.8% in 2009 to 22.1% in 2018), with a peak in 2012 (Figure 1). Most peaks occurred in the fall (Figure 2a).

#### 3.1.2. Serovar-Specific Trends, Seasonal Patterns, and Temporal Clusters

The most commonly isolated serovars from broiler breeder submissions were *S. enterica* serovars Kentucky (42.4% of 682 submissions), Heidelberg (19.2%), Typhimurium (5.4%), Enteritidis (3.7%), and I:Rough-O:r:1,2 (3.7%) (Table 3).

**Figure 3:**
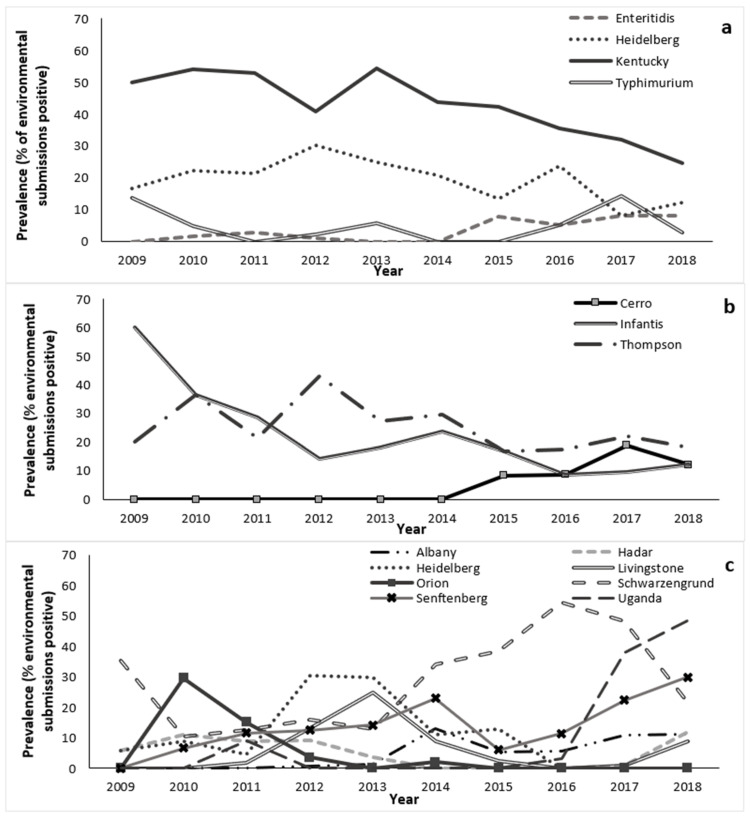
Trends in the prevalence of the most common *Salmonella enterica* serovars isolated from environmental samples submitted through the Ontario Hatchery and Supply Flock Policy between 2009 and 2018, by major poultry commodities. (**a**) Broiler breeders (*n* = 682 submissions); (**b**) Layer breeders (*n* = 195 submissions); and (**c**) Turkey breeders (*n* = 1368 submissions). A submission could be counted more than once because some submissions tested positive for more than one serovar.

Trends for the most common serovars are illustrated in Figure 3a. There was a steep, decreasing trend in the prevalence of *S. enterica* ser. Kentucky, especially after 2013; most of the *S. enterica* ser. Kentucky-positive submissions occurred in the fall and winter. There was a slightly decreasing trend in the prevalence of *S. enterica* ser. Heidelberg, with peaks in 2012 and 2016, and no apparent seasonal pattern. The prevalence of *S. enterica* ser. Typhimurium also generally decreased over the study period, with a peak in 2017. There was an increasing trend in the prevalence of *S. enterica* ser. Enteritidis, beginning in 2015.

A total of 48 serovars were isolated from broiler breeder submissions (Table 3). Of those, six were included in the temporal cluster detection analysis. Significant clusters were detected for the following *S. enterica* serovars: Enteritidis, Heidelberg, Kentucky, and Typhimurium (Table 4). Three of the four clusters were of long duration (≥6 months). The long-duration *S. enterica* serovars Heidelberg and Kentucky clusters each occurred during the first half of the study period, whereas the long-duration *S. enterica* ser. Enteritidis cluster occurred over 39 months during the latter half of the study period (May 2015 to July 2018) and included the majority (84% of 25 submissions) of the *S. enterica* ser. Enteritidis isolates. Conversely, the *S. enterica* ser. Typhimurium cluster was of short duration (<6 months) at the end of 2017 and included only 10 isolates.

### 3.2. Layer Breeders

#### 3.2.1. Prevalence, Trends, and Seasonal Patterns

There were 1848 submissions from layer breeders between 2009 and 2018 (Table 2). Of those, 119 (6.4%, 95% CI: 5.3 to 7.6%) tested positive for *S. enterica*. Overall, there was a decreasing trend in the submission-level prevalence during the study period (from 15.4% in 2009 to 4.9% in 2018) (Figure 1). Most peaks occurred in the fall, winter, or spring (Figure 2b).

#### 3.2.2. Serovar-Specific Trends, Seasonal Patterns, and Temporal Clusters

The most commonly isolated serovars from layer breeder submissions were *S. enterica* serovars Thompson (20.0% of 195 submissions) and Infantis (16.4%) (Table 3); *S. enterica* ser. Enteritidis was isolated infrequently (1.0%).

Trends for the most common serovars are illustrated in Figure 3b. The prevalence of *S. enterica* ser. Thompson fluctuated over the study period. There was a steep, decreasing trend in the prevalence of *S. enterica* ser. Infantis. *Salmonella enterica* ser. Cerro, accounting for 6.7% of submissions, was not detected among layer breeder submissions before 2015.

A total of 31 serovars were isolated from layer breeder submissions (Table 3). Of those, two were included in the temporal cluster detection analysis. A significant, long-duration cluster was detected for *S. enterica* ser. Infantis (Table 4); it occurred over 30 months during the first half of the study period.

### 3.3. Turkey Breeders

#### 3.3.1. Prevalence, Trends, and Seasonal Patterns

There were 3715 submissions from turkey breeders between 2009 and 2018 (Table 2). Of those, 1064 (28.6%, 95% CI: 27.2 to 30.1%) tested positive for *S. enterica*. Overall, there was an increasing trend in the submission-level prevalence during the study period (from 12.0% in 2009 to 24.5% in 2018), with a peak in 2015 (Figure 1). Most peaks occurred in the fall, winter, or summer (Figure 2c).

#### 3.3.2. Serovar-Specific Trends, Seasonal Patterns, and Temporal Clusters

The most commonly isolated serovars from turkey breeder submissions were *S. enterica* serovars Schwarzengrund (23.6% of 1368 submissions), Senftenberg (12.9%), Heidelberg (9.6%), Uganda (9.6%), Livingstone (5.9%), Orion (4.7%), Hadar (4.5%), and Albany (4.4%) (Table 3); *S. enterica* ser. Enteritidis was isolated infrequently (0.1%).

Trends for the most common serovars are illustrated in Figure 3c. There was an overall decreasing trend in the prevalence of *S. enterica* ser. Schwarzengrund, although the prevalence fluctuated widely, with a peak in 2016. The prevalence of *S. enterica* ser. Heidelberg also fluctuated greatly over the study period, with a peak in 2012–2013, and no isolates detected after 2015. There was an increasing trend in the prevalence of *S. enterica* ser. Senftenberg; most of the *S. enterica* ser. Senftenberg-positive submissions occurred in the fall. There was no apparent seasonal pattern for *S. enterica* serovars Schwarzengrund, Heidelberg, or Uganda.

A total of 69 serovars were isolated from turkey breeder submissions (Table 3). Of those, 13 were included in the temporal cluster detection analysis. Significant clusters were detected for all thirteen: *S. enterica* serovars Agona, Albany, Hadar, Heidelberg, Liverpool, Livingstone, Mbandaka, Newport, Orion, Schwarzengrund, Senftenberg, Typhimurium, and Uganda (Table 4). Eight of those had more than one cluster, which were spread throughout the study period. Except for the second or third clusters of *S. enterica* serovars Albany, Hadar, and Uganda, all significant clusters were of long duration. The primary long-duration clusters of *S. enterica* serovars Agona, Albany, Liverpool, Mbandaka, Orion, and Uganda included all, or most, of the isolates for those serovars.

## 4. Discussion

This study analyzed environmental data collected from 2009 to 2018 from Ontario’s poultry breeder flocks for the Ontario Hatchery and Supply Flock Policy monitoring program to determine the period prevalence, temporal trends and temporal clusters, and seasonal patterns of *S. enterica* for the major poultry commodities. The period prevalence in our study was higher than a baseline survey of *S. enterica* among turkey breeders in the European Union (13.6%) [20], which was conducted during a similar period and with similar methodology. Relative to the previous 11-year period (1998 to 2008) in Ontario [15], the period prevalence of *S. enterica* from environmental submissions was lower for broiler breeders (25.3% vs. 47.4% for the previous period) and layer breeders (6.4% vs. 25.7% for the previous period) and higher for turkey breeders (28.6% vs. 19.6% for the previous period). As no identifier was provided for the individual flocks sampled under the Ontario Hatchery and Supply Flock Policy during the current study period, flock-level prevalence could not be calculated and compared with the previous 11-year period. Descriptive analysis showed that the temporal trends of *S. enterica* prevalence between 2009 and 2018 varied among the major poultry commodities, with decreasing trends in prevalence for broiler breeders and layer breeders and an increasing trend for turkey breeders. Ontario is home to a primary turkey breeding and genetics company and is a global exporter of turkey genetics. To export hatching eggs, turkey breeder suppliers must follow environmental sampling schedules set by the importing country. In the European Union, additional sampling measures came into effect in 2010 as part of the National Control Programme for the Control of Salmonella in Turkey Flocks [21,22]. Thus, the increase in the period prevalence observed among turkey breeders in Ontario could be due, in part, to these additional sampling measures.

During the current study period (2009 to 2018), the period prevalence of *S. enterica* (without regard to serovar) was consistently higher in breeders (28.6%, 25.3%, and 6.4% in turkey, broiler, and layer breeders, respectively) than in the corresponding hatcheries (7.6%, 7.5%, and 1.6% in turkey, broiler, and layer hatcheries, respectively) [16]. This pattern (i.e., higher prevalence in breeder environmental samples than hatchery fluff samples) is consistent with the previous 11-year period (1998 to 2008) in Ontario [15,17]. This could be a combination of three key factors: exposure period; transmission; and control measures. *Salmonella enterica* can be introduced into a breeder flock at any point during the rearing and production stages, resulting in a long period of exposure during which a flock can become *S. enterica*-positive. *Salmonella enterica* can be transmitted horizontally and vertically. During a breeder flock’s lifespan, environmental sampling is performed at least three times (twice pre-lay and once during laying), whereas hatchery sampling will include offspring from any flock at least six times during their lay life. As the hatchery prevalence is consistently lower than the environmental prevalence detected among the breeder flocks and vertical transmission is only efficient for select serovars [10,11,23,24], many positive samples detected within the hatchery are likely due to horizontal transmission caused by random environmental contamination rather than exposure from the parent flocks [10,23,24,25]. Further, some intervention between breeding flocks and hatching is likely effective in reducing spread. These interventions could include biosecurity, sanitation, and screening measures after transport to the hatcheries [17]. Finally, different levels of analysis (submission-level prevalence from breeder environmental samples vs. sample-level prevalence from hatchery fluff samples) might have resulted in overestimation of the breeder-level estimates because submissions were considered *S. enterica*-positive even if only one of the environmental samples tested positive. Despite the lower prevalence at the hatchery level, *S. enterica* can be transmitted to lower levels of the production chain via contaminated chicks/poults. Thus, future research should focus on identifying risk factors for *S. enterica* at the breeder flock level and identifying potential sources of variation among flocks and across different levels of the poultry production chain to better understand the mechanics behind transmission between populations.

Retrospective scan statistics identified at least one temporal cluster for each major commodity. Clusters were identified for 4 of 6, 1 of 2, and 13 of 13 serovars investigated in broiler breeders, layer breeders, and turkey breeders, respectively. With few exceptions (*S. enterica* ser. Typhimurium in broiler breeders and non-primary clusters of *S. enterica* serovars Albany, Hadar, and Uganda in turkey breeders), most clusters were of long duration. Further, several of the primary long-duration clusters included all, or most (≥80%), of the isolates for the serovar, including *S. enterica* ser. Enteritidis in broiler breeders and *S. enterica* serovars Agona, Albany, Liverpool, Mbandaka, Orion, and Uganda in turkey breeders. The pervasiveness of long-duration clusters among breeder flocks could indicate a continuous common source [18], such as rodents, red mites, and lesser mealworm beetle larvae [12,13], persistently infected primary breeding birds, or, potentially, farm-to-farm transmission [18]. Alternatively, in turkey breeders, the more frequent sampling required for certification or export might explain, at least in part, the long-duration clusters observed. Finally, several of the primary long-duration clusters occurred near the end of the study period, including *S. enterica* ser. Enteritidis in broiler breeders and *S. enterica* serovars Albany, Liverpool, Mbandaka, Senftenberg, and Uganda in turkey breeders, indicating potential shifts in serovar dominance in these populations [17,18].

For several serovars, we noted temporal similarities between clusters from breeder environmental samples and clusters from hatchery fluff samples [16]. In broiler breeders, a relatively small cluster of *S. enterica* ser. Enteritidis isolates detected between May 2015 and July 2018 from environmental samples overlapped with a small cluster of *S. enterica* ser. Enteritidis isolates detected between October 2017 and February 2018 from fluff samples from broiler hatcheries [16]. Similar temporal overlaps were found for larger clusters of *S. enterica* serovars Heidelberg and Kentucky in broiler breeders and broiler hatcheries and *S. enterica* serovars Heidelberg, Newport, and Orion in turkey breeders and turkey hatcheries. Previous research conducted in Ontario between 1998 and 2008 [15,17] described similar breeder–hatchery-linked clusters for these commodities, including *S. enterica* serovars Hadar, Heidelberg, and Typhimurium in broiler breeders and broiler hatcheries and *S. enterica* ser. Heidelberg in turkey breeders and turkey hatcheries. The synchronicity of the clusters at these sequential stages of the production chain further supports that breeder flocks can be a source of contamination to the hatcheries via vertical and/or horizontal transmission, particularly for *S. enterica* ser. Heidelberg. Therefore, interventions targeted at breeder flocks could reduce the transmission of these serovars to the hatcheries and possibly reduce prevalence at the retail level.

*Salmonella enterica* ser. Schwarzengrund was the most commonly isolated serovar in our study, and it was isolated almost exclusively from turkey breeders. It was the most frequently isolated serovar from turkey breeder submissions (23.6%) and its prevalence increased sharply from 2013 to 2016. This is notably higher than the previous 11-year period (1998 to 2008) in Ontario (0.9%) [15]. One potential reason for the higher prevalence in our study relative to the previous period might be due to differences in sampling frequency, as the previous Ontario study was conducted before the implementation of the National Control Programme for the Control of Salmonella in Turkey Flocks in the European Union in 2010. Interestingly, *S. enterica* ser. Schwarzengrund has been identified only sporadically in Ontario’s hatcheries since 1998 [16,17]. This disparity in prevalence between the breeder and hatchery levels could be due to the serovar being less competent than other serovars at transferring vertically from parent to progeny. Notwithstanding, *S. enterica* ser. Schwarzengrund was the sixth most common serovar (5.8%) isolated from pooled fecal samples from commercial turkey flocks in Canada from 2013 to 2018 (with most of the positive samples coming from Ontario) [26], and it was one of 19 serovars associated with significantly higher rates of bacteremia in humans (compared with all non-typhoidal serovars) based on reports to the National Enteric Surveillance Program between 2006 and 2019 [27], emphasizing the importance of the recent increasing trend in Ontario’s turkey breeders.

Another prominent serovar in our study was *S. enterica* ser. Kentucky. It was isolated almost entirely from a single commodity—broiler breeders—and it was the most commonly isolated serovar from broiler breeder submissions (42.4%). This is consistent with the previous 11-year period (1998 to 2008) in Ontario, in which *S. enterica* ser. Kentucky was isolated almost exclusively from broiler breeders and accounted for 43.3% of the broiler breeder submissions [15]. *Salmonella enterica* ser. Kentucky is particularly capable of colonizing broiler chickens and is one of the most prominent serovars at all levels of Canada’s broiler production chain. It has been the most common serovar isolated from broiler fluff samples in Ontario’s hatcheries since 1998 [16,17], and it was the first, second, or third most common serovar identified in commercial broiler flocks in British Columbia, Alberta, Saskatchewan, Ontario, and Québec [26,28,29]. Further, several national studies have identified *S. enterica* ser. Kentucky as the most common serovar isolated from Canadian broiler carcasses at slaughter and retail chicken products [26,30,31]. Similarly, in the United States, *S. enterica* ser. Kentucky has remained the predominant serovar isolated from retail poultry products since 1998 [32,33].

Despite its high prevalence within all levels of the broiler production chain, relatively few cases of human salmonellosis are due to *S. enterica* ser. Kentucky [4,31,32,33,34,35,36], indicating that it is less competent than other serovars at infecting humans. Further, unlike *S. enterica* ser. Schwarzengrund, *S. enterica* ser. Kentucky was one of 20 serovars associated with significantly lower rates of bacteremia in humans (compared with all non-typhoidal serovars) [28], emphasizing that this serovar, while common in the broiler production chain, has not been a cause of serious illness in Canadians. However, of concern is the increase in antibiotic resistance in *S. enterica* ser. Kentucky isolates from broiler flock samples in Canada and internationally [22,26,29,30,37]. These studies highlight the importance of understanding the mechanisms by which *S. enterica* ser. Kentucky disseminates through the production chain to improve *Salmonella* control beyond antibiotic use. The steep, decreasing trend in the prevalence of this serovar coincides with the broiler breeder *Salmonella* vaccination program in Ontario, which began in January 2013 and includes *S. enterica* ser. Kentucky [38], indicating that a vaccination control program can be an effective alternative to antibiotics.

In our study, *S. enterica* ser. Heidelberg was the third most commonly isolated serovar, accounting for 11.8% of all positive submissions. It was the second most frequently isolated serovar from broiler breeders (19.2% of positive broiler breeder submissions), third most frequently isolated serovar from turkey breeders (9.6% of positive turkey breeder submissions), and it was isolated sporadically from layer breeders (1.5% of positive layer breeder submissions). This is divergent from the previous two decades in Ontario, during which *S. enterica* ser. Heidelberg was the most common serovar in environmental samples from broiler and turkey flocks from 1991 to 1998 [39] and in environmental samples from breeder flocks from 1998 to 2008 (broiler breeders: 40.9%; turkey breeders: 53.0%; and layer breeders: 31.0%) [15]. Although several long-duration temporal clusters of *S. enterica* ser. Heidelberg were identified in broiler breeders and turkey breeders in our study, most of them occurred during the early part of the study period. The decreasing trend in the prevalence of this serovar among broiler breeders coincides with the broiler breeder *Salmonella* vaccination program in Ontario, which began in January 2013 and includes *S. enterica* ser. Heidelberg [39]. The reduction in prevalence from previous periods, and during the current study period, indicates that improved and targeted control programs, biosecurity, sanitation, and/or screening measures have been successful in curtailing the spread of *S. enterica* ser. Heidelberg in Ontario’s poultry industry.

This study identified seasonal variation in *S. enterica* prevalence, although there was some inconsistency across poultry breeder commodities. In all commodities, peaks in prevalence frequently occurred in the fall, although peaks also often occurred in the winter or spring in layer breeders and in the winter or summer in turkey breeders. This is somewhat consistent with the previous 11-year period (1998 to 2008) in Ontario, in which there was an increased risk of *S. enterica* in the fall, depending on the year [15]. Among Ontario’s poultry hatcheries during the same period as our study (2009 to 2018), seasonal variation in *S. enterica* prevalence was also detected, with a higher risk in fluff samples submitted from turkey hatcheries in the summer and fall compared with samples submitted from broiler hatcheries in the winter [16]. In Canada and elsewhere, consistent seasonal patterns have not been observed throughout the broiler production chain. An Albertan monitoring program of mostly broiler breeder flocks [18], a Canadian study of broiler carcasses at slaughter and retail samples [40], and an American study of broiler samples collected at slaughter [41] showed no seasonal effects of *S. enterica* prevalence. Conversely, a Mexican study [42] identified increased prevalence in the winter in broiler retail samples, whereas Indian [43] and Korean [44] studies of broiler samples collected during slaughter identified increased prevalence in the summer. Due to the lack of consistent findings among studies, it is difficult to generalize about the effect of season on the prevalence of *S. enterica* other than to note that summer or fall tend to be associated with a higher risk at most levels of the production chain in Ontario.

Our data were collected at the population level, and interpretations are likely representative of the temporal trends and seasonal patterns of *S. enterica* within Ontario’s broiler, layer, and turkey breeder environments. One limitation of our study stemmed from the absence of flock identification. The lack of ability to control for clustering in the data could have resulted in an overestimation of the prevalence estimates, potentially limiting the conclusions that can be drawn. Thus, monitoring programs should include flock identification to ensure the accuracy of the results of data analysis. As in previous studies, identifying temporal trends and clusters of different serovars over the study period enabled us to observe temporal similarities between breeder flocks and hatcheries within a commodity. For example, clusters of *S. enterica* serovars Enteritidis, Heidelberg, Kentucky, Newport, and Orion in breeder flocks were temporally linked to clusters in their respective hatcheries. This suggests that breeder flocks continue to be a source of *S. enterica* to the hatcheries. Therefore, there is a need to determine the underlying reasons for the dominance of a select few serovars within the poultry production chain. This would enable the development of better management practices, biosecurity protocols, and sanitation programs for these and all *S. enterica* serovars. Future research should work towards identifying risk factors for *S. enterica* in breeding flocks and identifying sources of variation among flocks.

## Figures and Tables

**Table 1:** Ontario Hatchery and Supply Flock Policy environmental sampling schedule for Ontario poultry breeder flocks.

Age	Sample Type	Primary Certification Program	Multiplier Certification Program	Multiplier Monitoring Program	EU Compatible
Day old	Chick Box Papers	√	√	√	√
4 weeks of age	Boot Samples * Dust Pad Samples **	-	-	-	√
2–4 weeks before transfer or 16–24 weeks of age	Swab Samples ***	√	√	√	-
24–27 weeks of age and every 3 weeks thereafter	Boot Samples * Dust Pad Samples **	-	-	-	√
At least 30 days thereafter	Swab Samples ***	√	√	-	-
Once at peak of production	Swab Samples ***	-	-	√	-

* One sample per barn. ** Two samples per barn. *** One sample per 1000 birds in the flock or a minimum of three samples. Source: Ontario Ministry of Agriculture, Food, and Rural Affairs. Ontario Hatchery and Supply Flock Policy. 2021.

**Table 2:** Overall, submission-level, period prevalence of *Salmonella enterica* isolated from environmental samples submitted through the Ontario Hatchery and Supply Flock Policy between 2009 and 2018, by major poultry commodities.

Poultry Commodity	Number of Samples Submitted	Number (and Percentage) of Submissions	Number of *S. enterica*-Positive Submissions *	Submission-Level Prevalence (%)	95% Confidence Interval (%)
Broiler breeders	39,529	2446 (30.5)	619	25.3	23.6–27.0
Layer breeders	17,098	1848 (23.1)	119	6.4	5.3–7.6
Turkey breeders	51,747	3715 (46.4)	1064	28.6	27.2–30.1
**Overall**	**108,374**	**8009**	**1802**	**22.5**	**21.6–23.4**

* A submission was considered *S. enterica*-positive if at least one sample tested positive for *S. enterica*, regardless of the number of serovars isolated from the sample, and *S. enterica*-negative if none of the samples tested positive for *S. enterica*.

**Table 3:** Submission-level frequency of *Salmonella enterica* serovars isolated from environmental samples submitted through the Ontario Hatchery and Supply Flock Policy between 2009 and 2018, by major poultry commodities.

Serovar	Group	Broiler Breeders	Layer Breeders	Turkey Breeders	Total
Agona	B	7	2	34	43
Albany	C2	0	0	60	60
Anatum	E1	8	0	2	10
Bietri	N	1	0	0	1
Braenderup	C1	1	3	1	5
Brandenburg	B	0	0	1	1
Bredeney	B	0	0	3	3
Cerro	C1	0	**13**	0	13
Cubana	G	2	0	0	2
Derby	B	0	0	1	1
Enteritidis	D	**25**	2	2	29
Give	E1	0	0	3	3
Hadar	C2	10	4	61	75
Hartford	C1	2	0	2	4
Heidelberg	B	**131**	3	**132**	**266**
I:1,4,5,12:i:-	B	0	2	0	2
I:10:-:1,5	E1	0	0	2	2
I:10:EH:-	E1	0	0	1	1
I:10:I,z13:-	E1	0	0	5	5
I:4 12:-:1 7 4:-:7	B	0	0	1	1
I:4 12:-:1,7 4:-:1,7	B	0	0	1	1
I:4 12:I:- 4:I:-	B	0	1	0	1
I:4 12:R:-	B	1	0	0	1
I:4 12:R:- 4:R:-	B	1	0	0	1
I:4,12,:-:-	B	0	0	2	2
I:4,12:-:1,2	B	1	0	0	1
I:4,12:-:1,5	B	0	0	1	1
I:4,12:-:1,7	B	0	0	1	1
I:4,12:d:-	B	0	0	1	1
I:4,12:i:-	B	3	3	1	7
I:4,5,12:-:-	B	2	0	0	2
I:4,5,12:-:1,2	B	1	0	0	1
I:4,5,12:b:-	B	0	0	2	2
I:4,5,12:i:-	B	5	**15**	4	24
I:4,5:-:- 4:-:-	B	1	0	0	1
I:6,7,14:-:1,5	C1	0	0	1	1
I:6,7:-:-	C1	0	0	1	1
I:6,7:-:1,5	C1	0	2	0	2
I:6,7:-:I,w	C1	0	0	1	1
I:6,8:-:-	C2	0	0	2	2
I:8,20:i:-	C2	2	0	0	2
I:Rough-O:-:1 7 -:D:7	--	0	0	1	1
I:Rough-O:-:1,5	--	1	0	5	6
I:Rough-O:-:z6	--	1	0	0	1
I:Rough-O:3,h:1,2	--	0	0	1	1
I:Rough-O:D:-	--	1	0	1	2
I:Rough-O:D:1 7 -:D:7	--	1	0	0	1
I:Rough-O:D:1,7	--	0	0	11	11
I:Rough-O:D:L W -:D:L W	--	0	0	1	1
I:Rough-O:d:l,w	--	1	0	0	1
I:Rough-O:EH:-	--	0	0	2	2
I:Rough-O:EH:1 2	--	1	0	4	5
I:Rough-O:EH:1 2 -:EH:2	--	0	0	3	3
I:Rough-O:EH:1 5 -:EH:5	B	0	0	5	5
I:Rough-O:EH:1,5	--	0	0	1	1
I:Rough-O:f,g,s:-	--	0	0	1	1
I:Rough-O:g,s,t:-	--	0	0	2	2
I:Rough-O:I,z13:1,5	--	0	0	1	1
I:Rough-O:i:-	--	0	1	0	1
I:Rough-O:i:1,2	--	0	0	1	1
I:Rough-O:i:z6	--	6	0	0	6
I:Rough-O:k:1,5	--	1	1	1	3
I:Rough-O:r:-	B	2	0	0	2
I:Rough-O:r:1,2	--	**25**	1	8	34
I:Rough-O:r:1,5	--	1	0	0	1
I:Rough-O:z10:e,n,x	--	0	0	2	2
I:Rough-O:z10:e,n,z15	--	1	0	0	1
I:Rough-O:z29:-	--	0	1	0	1
I:Rough-O:z4,z24:-	--	0	0	8	8
Indiana	B	1	0	0	1
Infantis	C1	9	**32**	9	50
Kedougou	G	1	**11**	2	14
Kentucky	C2	**289**	7	1	**297**
Kiambu	B	14	0	0	14
Litchfield	C2	3	0	1	4
Liverpool	E4	0	3	22	25
Livingstone	C1	24	7	**81**	112
Mbandaka	C1	14	6	39	59
Montevideo	C1	2	1	3	6
Muenchen	C2	0	0	9	9
Muenster	E1	0	0	10	10
Newport	C2	0	4	27	31
Ohio	C1	0	1	0	1
Oranienburg	C1	5	0	1	6
Orion	E2	7	5	64	76
Ouakam	D2	0	8	6	14
Putten	G	8	0	0	8
Schwarzengrund	B	8	3	**323**	**334**
Senftenberg	E4	8	7	**176**	**191**
Saintpaul	B	0	0	10	10
Tennessee	C1	2	0	16	18
Thompson	C1	1	**39**	8	48
Typhimurium	B	**37**	6	39	82
Uganda	E1	0	0	**132**	**132**
Virchow	C1	0	1	0	1
Westhampton	E1	0	0	1	1
Worthington	G2	3	0	0	3
Total *		**682**	**195**	**1368**	**2245**

Within a column, the serovars with the highest frequency are shown in bold. * The discrepancy between the total number of submissions reported in Table 2 and Table 3 results from some submissions having tested positive for more than one serovar. -- Serovars unable to be grouped.

**Table 4:** Temporal clusters of *Salmonella enterica* serovars isolated from environmental samples submitted through the Ontario Hatchery and Supply Flock Policy between 2009 and 2018, by major poultry commodities (*p* ≤ 0.05).

Poultry Commodity	Serovar ^+^	First Cluster ^++^	Second Cluster ^++^	Third Cluster ^++^
Broiler breeders	Enteritidis (25)	2015/5–2018/7 ^#^ (21)		
	Heidelberg (131)	2011/8–2013/12 (52)		
	Kentucky (289)	2009/9–2013/12 (159)		
	Typhimurium (37)	2017/9–2017/12 (10)		
Layer breeders	Infantis (32)	2009/1–2011/6 (14)		
Turkey breeders	Agona (34)	2010/8–2013/3 (28)	2015/9–2016/5 (6)	
	Albany (60)	2014/1–2018/11 (57)	2012/12–2013/3 (3)	
	Hadar (61)	2010/2–2012/8 (38)	2018/9–2018/12 (14)	
	Heidelberg (132)	2012/4–2013/9 (83)	2014/10–2015/11 (27)	2009/5–2013/10 (21)
	Liverpool (22)	2015/4–2018/10 (22)		
	Livingstone (81)	2012/11–2013/10 (44)	2011/12–2012/6 (11)	
	Mbandaka (39)	2015/2–2018/11 (39)		
	Newport (27)	2017/4–2017/9 (12)	2010/12–2011/6 (6)	
	Orion (64)	2010/2–2011/11 (57)	2012/2–2012/10 (5)	
	Schwarzengrund (323)	2015/4–2017/11 (178)		
	Senftenberg (176)	2018/7–2018/12 (32)		
	Typhimurium (39)	2015/6–2016/3 (10)		
	Uganda (132)	2017/2–2018/12 (118)	2011/6–2011/12 (10)	2016/9–2016/12 (4)

Iterative temporal scan performed using SaTScan v9 (Kulldorff, 2018). The scanning window size was 50% of the study period. ^+^ Total number of isolates for each serovar. ^++^ Number of isolates within the cluster. ^#^ Dates are given as year/month. A submission could be counted more than once because some submissions tested positive for more than one serovar.

## Data Availability

All relevant data are included in the manuscript.

## References

[1] Government of Canada (2022). Reported Cases from 1924 to 2019 in Canada—Notifiable Diseases On-Line.

[2] Government of Ontario (2022). Reportable Disease Trends in Ontario.

[3] Emond-Rheault J.-G., Jeukens J., Freschi L., Kukavica-Ibrulj I., Boyle B., Dupont M.-J., Colavecchio A., Barrere V., Cadieux B., Arya G. (2017). A Syst-OMICS Approach to Ensuring Food Safety and Reducing the Economic Burden of Salmonellosis. Front. Microbiol..

[4] Government of Canada (2017). Foodnet Canada Annual Report 2017.

[5] Varga C., Pearl D.L., McEwen S.A., Sargeant J.M., Pollari F., Guerin M.T. (2013). Incidence, Distribution, Seasonality, and Demographic Risk Factors of *Salmonella* Enteritidis Human Infections in Ontario, Canada, 2007–2009. BMC Infect. Dis..

[6] Middleton D., Savage R., Tighe M.K., Vrbova L., Walton R., Whitfield Y., Varga C., Lee B., Rosella L., Dhar B. (2014). Risk Factors for Sporadic Domestically Acquired *Salmonella* Serovar Enteritidis Infections: A Case-control Study in Ontario, Canada, 2011. Epidemiol. Infect..

[7] Snyder T.R., Boktor S.W., M’ikanatha N.M. (2019). Salmonellosis Outbreaks by Food Vehicle, Serotype, Season, and Geographical Location, United States, 1998 to 2015. J. Food Prot..

[8] Marus J.R., Magee M.J., Manikonda K., Nichols M.C. (2019). Outbreaks of *Salmonella enterica* Infections Linked to Animal Contact: Demographic and Outbreak Characteristics and Comparison to Foodborne Outbreaks—United States, 2009–2014. Zoonoses Public Health.

[9] Anderson T.C., Marsden-Haug N., Morris J.F., Culpepper W., Bessette N., Adams J.K., Bidol S., Meyer S., Schmitz J., Erdman M.M. (2017). Multistate Outbreak of Human *Salmonella* Typhimurium Infections Linked to Pet Hedgehogs—United States, 2011–2013. Zoonoses Public Health.

[10] Collineau L., Phillips C., Chapman B., Agunos A., Carson C., Fazil A., Reid-Smith R.J., Smith B.A. (2020). A within-Flock Model of *Salmonella* Heidelberg Transmission in Broiler Chickens. Prev. Vet. Med..

[11] Varga C., Middleton D., Walton R., Savage R., Tighe M.-K., Allen V., Ahmed R., Rosella L. (2012). Evaluating Risk Factors for Endemic Human *Salmonella* Enteritidis Infections with Different Phage Types in Ontario, Canada Using Multinomial Logistic Regression and a Case-Case Study Approach. BMC Public Health.

[12] Davies R.H., Wales A.D. (2014). Developments in *Salmonella* Control in Eggs. Adv. Microb. Food Saf..

[13] Jensen A.N., Dalsgaard A., Stockmarr A., Nielsen E.M., Baggesen D.L. (2006). Survival and Transmission of *Salmonella enterica* Serovar Typhimurium in an Outdoor Organic Pig Farming Environment. App. Environ. Microbiol..

[14] Government of Ontario (2021). Ontario Hatchery and Supply Flock Policy.

[15] Sivaramalingam T., McEwen S.A., Pearl D.L., Ojkic D., Guerin M.T. (2013). A Temporal Study of *Salmonella* Serovars from Environmental Samples from Poultry Breeder Flocks in Ontario between 1998 and 2008. Can. J. Vet. Res..

[16] Murray C.E., Varga C., Ouckama R., Guerin M.T. (2022). Temporal Study of *Salmonella enterica* Serovars Isolated from Fluff Samples from Ontario Poultry Breeder Flocks between 2009 and 2018. Pathogens.

[17] Sivaramalingam T., Pearl D.L., McEwen S.A., Ojkic D., Guerin M.T. (2013). A Temporal Study of *Salmonella* Serovars from Fluff Samples from Poultry Breeder Hatcheries in Ontario between 1998 and 2008. Can. J. Vet. Res..

[18] Guerin M.T., Martin S.W., Darlington G.A., Rajic A. (2005). A Temporal Study of *Salmonella* Serovars in Animals in Alberta between 1990 and 2001. Can. J. Vet. Res..

[19] Kulldorff M. (2018). SaTScan User Guide.

[20] European Parliament (2008). Report of the Task Force on Zoonoses Data Collection on the Analysis of the Baseline Survey on the Prevalence of Salmonella in Turkey Flocks, in the EU, 2006–2007.

[21] European Parliament (2008). Implementing Regulation (EC) No 2160/2003 of the European Parliament and of the Council as Regards a Community Target for the Reduction of the Prevalence of *Salmonella* Enteritidis and *Salmonella* Typhimurium in Turkeys. Off. J. Eur. Union.

[22] Government of the United Kingdom *Salmonella* in Livestock Production in Great Britain. https://www.gov.uk/government/publications/Salmonella-in-livestock-production-in-great-britain.

[23] Barrow P.A. (2000). The Paratyphoid *Salmonellae*. Rev. Scient. Technq. Int. Off. Epizoot..

[24] Braden C.R. (2006). *Salmonella enterica* Serotype Enteritidis and Eggs: A National Epidemic in the United States. Clin. Infect. Dis..

[25] Liljebjelke K.A., Hofacre C.L., Liu T., White D.G., Agers S., Young S., Maurer J.J. (2005). Vertical and Horizontal Transmission of *Salmonella* within Integrated Broiler Production System. Foodborne Pathog. Dis..

[26] Caffrey N., Agunos A., Gow S., Liljebjelke K., Mainali C., Checkley S.L. (2021). Prevalence and Antimicrobial Resistance in Broiler Chicken and Turkey Flocks in Canada from 2013 to 2018. Zoonoses Public Health.

[27] Tamber S., Dougherty B., Nguy K. (2021). *Salmonella enterica* Serovars Associated with Bacteremia in Canada, 2006–2019. Can. Commun. Dis. Rep..

[28] Diarra M.S., Delaquis P., Rempel H., Bach S., Harlton C., Aslam M., Pritchard J., Topp E. (2014). Antibiotic Resistance and Diversity of *Salmonella enterica* Serovars Associated with Broiler Chickens. Food Prot..

[29] Agunos A., Arsenault R.K., Avery B.P., Deckert A.E., Gow S.P., Janecko N., Léger D.F., Parmley E.J., Reid-Smith R.J., McEwen S.A. (2018). Changes in Antimicrobial Resistance Levels among *Escherichia coli*, *Salmonella*, and *Campylobacter* in Ontario Broiler Chickens between 2003 and 2015. Can. J. Vet. Res..

[30] Romero-Barrios P., Deckert A., Parmley E.J., Leclair D. (2020). Antimicrobial Resistance Profiles of *Escherichia coli* and *Salmonella* Isolates in Canadian Broiler Chickens and Their Products. Foodborne Pathog. Dis..

[31] Government of Canada (2018). Foodnet Canada Annual Report 2018.

[32] Shah D.H., Paul N.C., Sischo W.C., Crespo R. (2017). Guard, Population Dynamics and Antimicrobial Resistance of the Most Prevalent Poultry-Associated *Salmonella* Serotypes. Poult. Sci..

[33] Government of the United States of America (2015). Serotypes Profile of Salmonella Isolates from Meat and Poultry Products.

[34] Government of the United States of America (2013). An Atlas of Salmonella in the United States, 1968–2011.

[35] Government of Canada (2016). Foodnet Canada Annual Report 2016.

[36] Government of Canada (2013). Foodnet Canada Annual Report 2013.

[37] Mulvey M.R., Boyd David A., Finley R., Fakharuddin K., Langner S., Allen V., Ang L., Bekal S., El Bailey S., Haldane D. (2013). Ciprofloxacin-resistant *Salmonella enterica* Serovar Kentucky in Canada. Emerg. Infect. Dis..

[38] Murray C.E. (2022). A Temporal Study of *Salmonella enterica* Serovars in Poultry Breeder Flocks and Hatcheries in Ontario from 2009 to 2018. MSc. Thesis.

[39] Zhang X., McEwen B., Mann E., Martin W. (2005). Detection of Clusters of *Salmonella* in Animals in Ontario from 1991 to 2001. Can. Vet. J..

[40] Smith B.A., Meadows S., Meyers R., Parmley E.J., Fazil A. (2019). Seasonality and Zoonotic Foodborne Pathogens in Canada: Relationships between Climate and *Campylobacter*, *E. coli* and *Salmonella* in Meat Products. Epidemiol. Infect..

[41] Parveen S., Taabodi M., Schwarz J.G., Oscar T.P., Harter-Dennis J., White D.G. (2007). Prevalence and Antimicrobial Resistance of *Salmonella* Recovered from Processed Poultry. J. Food Prot..

[42] Regalado-Pineda I.D., Rodarte-Medina R., Resendiz-Nava C.N., Saenz-Garcia C.E., Castañeda-Serrano P., Nava G.M. (2020). Three-year longitudinal study: Prevalence of *Salmonella enterica* in Chicken Meat is Higher in Supermarkets than Wet Markets from Mexico. Foods.

[43] Bhuvaneswa M., Shanmughap S., Natarajase K. (2015). Prevalence of Multidrug-Resistant (MDR) *Salmonella enteritidis* in Poultry and Backyard Chicken from Tiruchirappalli, India. Microbiol. J..

[44] Lee S.K., Choi D., Kim H.S., Kim D.H., Seo K.H. (2016). Prevalence, Seasonal Occurrence, And Antimicrobial Resistance of *Salmonella* spp. Isolates Recovered from Chicken Carcasses Sampled at Major Poultry Processing Plants of South Korea. Foodborne Pathog. Dis..

